# Supplementary material for: NephroPOC - Risk assessment and prediction of acute kidney injury in emergency patients with suspected organ dysfunction: Secondary analysis from the prospective observational LifePOC study
Source: PLoS One. 2026 Feb 18;21(2):e0339763. doi: 10.1371/journal.pone.0339763 (PMC12915921; doi:10.1371/journal.pone.0339763)
Supplement: S1 Text — (DOCX) [file pone.0339763.s001.docx]

# Supplemental digital content to

NephroPOC - Risk assessment and prediction of acute kidney injury in emergency patients with suspected organ dysfunction: secondary analysis from the prospective observational LifePOC study

Caroline Neumann¹, Margit Leitner¹, Thomas Lehmann², Michael Kiehntopf^³^, Michael Joannidis^⁴^, Myrto Bolanaki^⁵^, Anna Slagman^⁵^, Martin Möckel^⁵^, Michael Bauer¹ and Johannes Winning¹

¹Department of Anaesthesiology and Intensive Care Medicine, Jena University Hospital, Am Klinikum 1, 07747 Jena, Germany

²Centre for Clinical Studies, Jena University Hospital, Am Klinikum 1, 07747 Jena, Germany

^³^Institute of Clinical Chemistry and Laboratory Diagnostics and Integrated Biobank Jena (IBBJ), Jena University Hospital, Am Klinikum 1, 07747 Jena, Germany

^⁴^Division of Intensive Care and Emergency Medicine, Department of Internal Medicine, Medical University Innsbruck, Anichstrasse 35, 6020 Innsbruck, Austria

^⁵^Department of Emergency and Acute Medicine, Campus Virchow and Mitte, Charité-Universitätsmedizin Berlin, Augustenburger Platz 1, 13353 Berlin, Germany

# Appendix A – Extended methods

## Measurement of biomarkers and estimation of GFR

After study inclusion, blood samples were collected within 12 hours after initial ED presentation and processed and stored as described previously (1). PenKid and bio-ADM were measured in EDTA plasma samples using immunoluminometric sphingotest® assays (SphingoTec GmbH, Hennigsdorf, Germany) as detailed in (2, 3). The 97.5^th^ percentile for penKid in healthy adult subjects is 89 pmol/L (90% CI 85 – 118 pmol/L) and 29 pg/mL (90% CI 27 – 38 pg/mL) for bio-ADM. The upper normal range of penKid (89 pmol/L) is also the clinical cut-off for diagnosis of AKI. MR-proADM and PCT were measured in EDTA plasma using commercially available automated immunofluorescent assays (MR-proADM™ KRYPTOR™ and PCT™ sensitive KRYPTOR™, B·R·A·H·M·S GmbH, Hennigsdorf, Germany). The 97.5^th^ percentiles reported in healthy individuals are 0.55 nmol/L for MR-proADM and 0.064 μg/l for PCT. DPP-3 was measured in EDTA plasma samples by 4TEEN4 Pharmaceuticals (Hennigsdorf, Germany) using the immunoluminometric assay sphingotest® DPP3 (SphingoTec GmbH, Hennigsdorf, Germany) as documented in the literature (4). The 97.5^th^ percentile for sphingotest® DPP3 in healthy adult subjects is 22 ng/mL (90% CI 18 – 34 ng/mL). The clinical cut-off for critically ill patients is 40 ng/mL (5, 6). Scr was determined on admission and during the further inpatient course (on ward or ICU) in the local in-clinic laboratories by the Jaffé method using the Abbot® system in Jena and the Roche® system in Berlin. Serum/plasma reference values were determined by Abbott® to range from 63.6 to 110.5 µmol/L (0.72-1.26 mg/dL) in men and 50.4 to 98.1 µmol/L (0.57-1.12 mg/dL) in women (in normal healthy adults 18 years of age or older) and 62-106 µmol/L (0.70-1.20 mg/dL) in men and 44-80 µmol/L (0.50-0.91 mg/dL) in women by the Roche® system. The two systems differ regarding the detection limit for Scr, which is 4.5 µmol/L (0.05 mg/dL) in the Abbot® system and 15 µmol/L (0.17 mg/dL) in the Roche® system. However, none of the patients had Scr values so low that they could not be detected with both systems. For biomarker measurements samples were blinded regarding clinical and demographic data of the patients.

GFR was estimated using the latest CKD-EPI formula based on age, Scr and gender. We also applied a formula recently developed by Beunders (7). In contrast to the routinely used creatinine-based equations, such as MDRD (“Modification of Diet in Renal Disease”), 2009 CKD-EPI (“Chronic Kidney Disease Epidemiology Collaboration”), and 2021 CKD-EPI, Beunders and colleagues developed a GFR formula using penKid, Scr and age (but not gender and race). We compared the performance of the different equations regarding prediction of AKI within 24, 48 and 72 hours after ED admission.

# Appendix B – Supplementary Results

**Table S1:** Baseline characteristics of patients at admission with and without known AKI status and main outcomes within 48 h (n=1434).

| **Patient characteristics** | **n** | **Total**  **(n=1434, 100%)** | **Patients with known AKI status**  **(n= 440, 30.7%)** | **Patients unknown AKI status**  **(n=994, 69.3%)** | **p-value** |
| --- | --- | --- | --- | --- | --- |
| Age (years) | 1434 | 72 [61-80] | 73 [63-79] | 72 [59-80] | 0.137 |
| Males, no. (%) | 1434 | 805 (56.1) | 251 (57.0%) | 554 (55.7%) | 0.686 |
| **Severity scores** |  |  |  |  |  |
| qSOFA | 1434 |  |  |  | **0.001** |
| 1 |  | 1164 (81.2%) | 333 (75.7%) | 831 (83.6%) |  |
| 2 |  | 250 (17.4%) | 102 (23.2%) | 148 (14.9%) |  |
| 3 points |  | 16 (1.1%) | 4 (0.9%) | 12 (1.2%) |  |
| Charlson Comorbidity Index | 1049 | 0 [1-3] | 2 [1-3] | 1 [0-3] | **<0.001** |
| CKD | 1049 | 82 (5.7%) | 40 (9.1%) | 42 (4.2%) | **0.001** |
| SOFA | 671 | 2 [1-4] | 3 [2-5] | 2 [1-3] | **<0.001** |
| Renal SOFA | 1414 | 1 [0-1] | 1 [0-1] | 0 [0-1] | **<0.001** |
| Body temperature (°C) | 1109 | 37.3 [36.8-38.0] | 37.4 [36.8-38.3] | 37.2 [36.8-37.9] | **0.005** |
| Heart rate (bpm) | 1402 | 90 [77-106] | 95 [81-110] | 88 [76-104] | **<0.001** |
| MAP (mmHg) | 1411 | 86 [72-100] | 83 [69-100] | 87 [73-99] | **0.041** |
| RR (per minute) | 1426 | 24 [22-27] | 25 [22-28] | 23 [22-26] | **<0.001** |
| paO2/FiO2 ratio | 1352 | 348 [286-457] | 333 [261-410] | 376 [295-457] | **<0.001** |
| GCS | 1424 | 15 [15-15] | 15 [15-15] | 15 [15-15] | 0.288 |
| **Laboratory variables and biomarkers** |  |  |  |  |  |
| pH | 1254 | 7.4 [7.36-7.44] | 7.4 (7.35-7.45) | 7.4 (7.36-7.44) | 0.779 |
| SBE (mmol/l) | 1254 | 0.8 [-1.6-  3.1] | -0.1 [-3.15-2.55] | 1.1 [-1.0-3.2] | **<0.001** |
| Bilirubin (μmol/l) | 731 | 0.59 [0.36-1.06] | 0.67 [0.41-1.12] | 0.57 [0.35-0.95] | **0.028** |
| Lactate (mmol/l) | 1273 | 1.7 [1.21-2.42] | 1.9 [1.32-2.7] | 1.65 [1.2-2.31] | **<0.001** |
| CRP (mg/l) | 1414 | 34.2 [7.2-106.8] | 70.9 [18.3-177.6] | 22.95 [5.0-80.5] | **<0.001** |
| Leukocytes (Gpt/l) | 1417 | 10.2 [7.5-14.0] | 11.7 [8.5-15.7] | 9.5 [7.2-13.3] | **<0.001** |
| Thrombocytes (Gpt/l) | 1418 | 232 [181-302] | 229 [167-308] | 232 [185-301] | 0.329 |
| **Biomarkers** |  |  |  |  |  |
| Scr (mg/dl) | 1434 | 1.02 [0.8-1.46] | 1.24 [0.88-1.85] | 0.97 [0.77-1.29] | **<0.001** |
| penKid (pmol/l) | 1434 | 64.21 [44.37-98.27] | 79.38 [49.4-126.76] | 59.86 [42.33-87.3] | **<0.001** |
| MR-pro-ADM (nmol/l)) | 1434 | 1.21 [0.79-2.04] | 1.82 [1.11-3.28] | 1.04 [0.72-1.7] | **<0.001** |
| bio-ADM (pg/mL) | 1434 | 43.71 [28.32-78.5] | 63.37 [36.7-121.52] | 38.99 [25.27-64.67] | **<0.001** |
| DPP-3 (ng/mL) | 440 | 18.38 [12.25-31.41] | 18.69 [12.51-32.66] | 18.17 [12.19-31.19] | 0.295 |
| PCT (μg/l) | 1434 | 0.14 [0.08-0.51] | 0.32 [0.11-2.2] | 0.11 [0.07-0.28] | **<0.001** |
| **Admission diagnosis** | 1434* |  |  |  |  |
| Pulmonary diseases |  | 512 (35.7%) | 169 (38.4%) | 343 (34.5%) | 0.087 |
| Cardiovascular diseases |  | 337 (23.5%) | 112 (25.5%) | 225 (22.6%) | 0.137 |
| Sepsis |  | 139 (9.7%) | 76 (17.3%) | 63 (6.3%) | **<0.001** |
| Diseases of UGT |  | 217 (15.1%) | 95 (21.6%) | 122 (12.3%) | **<0.001** |
| Diseases of digestive system |  | 106 (7.4%) | 41 (9.3%) | 65 (6.5%) | **0.042** |
| Solid tumours |  | 25 (1.7%) | 11 (2.5%) | 14 (1.4%) | 0.110 |
| Others |  | 435 (30.3%) | 118 (26.8%) | 317 (31.9%) | 0.977 |
| **Focus of infection within 48 h** | 1434 |  |  |  |  |
| Pulmonary |  | 530 (37.0%) | 174 (39.5%) | 356 (35.8%) | 0.099 |
| Intraabdominal |  | 117 (8.2%) | 46 (10.5%) | 71 (7.1%) | 0.167 |
| Urogenital |  | 121 (8.4%) | 58 (13.2%) | 63 (6.3%) | **<0.001** |
| Cardiovascular |  | 6 (0.4%) | 5 (1.1%) | 1 (0.1%) | **0.012** |
| Cerebral |  | 6 (0.4 %) | 5 (1.1%) | 3 (0.3%) | **0.024** |
| Skin and soft tissue |  | 46 (3.2%) | 20 (4.5%) | 26 (2.6%) | **0.043** |
| Primary bacteraemia |  | 5 (0.3%) | 4 (0.9%) | 1 (0.1%) | **0.033** |
| Others |  | 24 (1.7%) | 7 (1.6%) | 17 (1.7%) | 0.621 |
| Unknown |  | 81 (5.6%) | 25 (5.7%) | 56 (5.6%) | 0.529 |
| None |  | 498 (34.7%) | 98 (22.3%) | 400 (40.2%) | 1.000 |
| **Secondary outcomes within 48 h** | 1434 |  |  |  |  |
| Sepsis |  | 399 (28.7%) | 228 (52.5%) | 171 (17.8%) | **<0.001** |
| Septic shock |  | 49 (3.5%) | 40 (9.2%) | 9 (0.9%) | **<0.001** |
| Vasopressors/inotropes |  | 98 (6.8%) | 79 (18.0%) | 19 (1.9%) | **<0.001** |
| Mechanical ventilation |  | 121 (8.4%) | 72 (16.4%) | 49 (4.9%) | **<0.001** |
| RRT |  | 18 (1.3%) | 16 (3.6%) | 2 (0.2%) | **<0.001** |

**Abbreviations:** qSOFA: quick Sequential Organ Failure Assessment; CKD: chronic kidney disease; SOFA: Sequential Organ Failure Assessment; MAP: mean arterial pressure; RR: respiratory rate; GCS: Glasgow Coma Scale; SBE: standard base excess; Scr: serum creatinine; CRP: C-reactive protein; penKid: proenkephalin A 119-159; MR-pro-ADM: midregional proadrenomedullin; bio-ADM: bioactive adrenomedullin; DPP-3: dipeptidylpeptidase-3 ; PCT: procalcitonin; UGT: urogenital tract; RRT: renal replacement therapy; For continuous variables median [25^th^-75^th^ percentile] and for categorical variables absolute and (relative) frequencies are reported. * In some patients more than one admission diagnosis was recorded.

**Table S2:** Organ support within 48 hours and endpoints within 28 days of patients with and without AKI within 48 h

|  | **AKI (n=83)** | **No AKI (n=357)** | **p-value** |
| --- | --- | --- | --- |
| **Organ support within 48 h** | | | |
| **RRT** | 11 (13.3%) | 5 (1.4%) | **<0.001** |
| **Vasopressors/inotropes** | 24 (28.9%) | 55 (15.4%) | **0.007** |
| **Mechanical ventilation** | 23 (27.7%) | 49 (13.7%) | **0.005** |
| **Endpoints within 28 days** | | | |
| **LOS ICU (days)** | 7.5 [2-14] | 4 [2-8] | 0.044 |
| **LOS hospital (days)** | 11.5 [7-28] | 10 [7-18] | 0.271 |
| **28-day mortality** | 14/82* (17.1%) | 36/351* (10.3%) | **0.006** |

**Abbreviations:** RRT: renal replacement therapy; LOS: length of stay; ICU: intensive care unit. * Due to missing mortality data for 6 patients in the non-AKI group and 1 patient in the AKI group.

**Table S3:** estimated GFR calculated from the biomarker values of patients at admission with and without AKI within 48 hours (n=440)

| **Patient characteristics** | **Total**  **(n=440, 100%)** | **Patients with AKI**  **(n= 83,18.9%)** | **Patients without AKI**  **(n=357, 81.1%)** | **p-value** |
| --- | --- | --- | --- | --- |
| eGFR CKD-EPI | 53.58 [32.42-84.01] | 47.09 [28.93-65.37] | 57.88 [35.04-86.36] | 0.003 |
| eGFR PENK-Crea | 58.79 [34.99-84.89] | 46.16 [26.68-65.79] | 64.19 [38.04-89.49] | 0.001 |
| eGFR MDRD | 52.93 [33.12-82.11] | 46.51 [29.87-65.1] | 56.93 [34.32-84.57] | 0.005 |

**Abbreviations:** AKI: acute kidney injury; GFR: glomerular filtration rate

**Table S4:** Prediction of AKI within 24, 48, and 72 h using admission penKid, Scr and eGFR

| **Model** | **n** | **Events** | **Model Chi2** | **p-value** | **AUCROC [95% CI]** |
| --- | --- | --- | --- | --- | --- |
| **24 h** | | | | | |
| penKid | 755 | 53 | 39.8 | <0.00001 | 0.725 [0.653,0.797] |
| sCr | 755 | 53 | 21.6 | <0.00001 | 0.706 [0.633,0.778] |
| eGFR PENK-Crea | 755 | 53 | 29.4 | <0.00001 | 0.717 [0.643,0.79] |
| eGFR CKD-EPI | 755 | 53 | 23.4 | <0.00001 | 0.692 [0.618,0.767] |
| eGFR MDRD | 755 | 53 | 17.9 | 0.00002 | 0.696 [0.62,0.772] |
| **48h** | | | | | |
| penKid | 440 | 83 | 23.5 | <0.00001 | 0.662 [0.599,0.724] |
| sCr | 440 | 83 | 4.9 | 0.02742 | 0.596 [0.529,0.663] |
| eGFR PENK-Crea | 440 | 83 | 16.3 | 0.00005 | 0.640 [0.577,0.704] |
| eGFR CKD-EPI | 440 | 83 | 9.5 | 0.00205 | 0.605 [0.540,0.670] |
| eGFR MDRD | 440 | 83 | 3.8 | 0.05293 | 0.599 [0.532,0.667] |
| **72 h** | | | | | |
| penKid | 328 | 104 | 13.8 | 0.00021 | 0.626 [0.563,0.689] |
| sCr | 328 | 104 | 0.02 | 0.8984 | 0.521 [0.454,0.589] |
| eGFR PENK-Crea | 328 | 104 | 6.6 | 0.0111 | 0.588 [0.523,0.653] |
| eGFR CKD-EPI | 328 | 104 | 1.8 | 0.1798 | 0.544 [0.478,0.61] |
| eGFR MDRD | 328 | 104 | 0.0 | 0.9898 | 0.531 [0.47,0.604] |

**Abbreviations:** AKI: acute kidney injury; penKid: Proenkephalin A 119-159; Scr: serum creatinine; GFR: glomerular filtration rate

**Table S5:** Prediction of AKI within 48h using admission biomarkers and GFR estimates: Net reclassification improvement based on GFR categories.

| **Patients without AKI within 48** **h** | | | | | | **Patients with AKI within 48 h** | | | | | |
| --- | --- | --- | --- | --- | --- | --- | --- | --- | --- | --- | --- |
|  |  | **PENK-Crea** | | | |  |  | **PENK-Crea** | | | |
|  |  | **(0,30]** | **(30,60]** | **(60,90]** | **(90,140]** |  |  | **(0,30]** | **(30,60]** | **(60,90]** | **(90,140]** |
| **CKD-EPI** | **(0,30]** | 65 | 15 | 0 | 0 | **CKD-EPI** | **(0,30]** | 25 | 1 | 0 | 0 |
|  | **(30,60]** | 7 | 74 | 30 | 3 |  | **(30,60]** | 5 | 29 | 4 | 0 |
|  | **(60,90]** | 0 | 9 | 62 | 25 |  | **(60,90]** | 0 | 4 | 9 | 1 |
|  | **(90,140]** | 0 | 1 | 12 | 62 |  | **(90,140]** | 0 | 0 | 5 | 5 |
|  |  |  |  |  |  |  |  |  |  |  |  |
|  | **Patients with lower GFR in PENK-Crea: n=29** | | | | |  | **Patients with lower GFR in PENK-Crea: n=14** | | | | |
|  |  |  |  |  |  |  |  |  |  |  |  |
|  | **Patients with higher GFR in PENK-Crea: n=73** | | | | |  | **Patients with higher GFR in PENK-Crea: n=6** | | | | |
|  |  |  |  |  |  |  |  |  |  |  |  |
|  | **Net gain: n=44** | |  |  |  |  | **Net gain: n=8** | |  |  |  |
|  | **Rel. gain: 12.1%** | |  |  |  |  | **Rel. gain: 9.1%** | |  |  |  |

**Table S6:** AUC of biomarkers to predict AKI within 48 hours in the whole cohort and stratified by treatment unit (n=440)

| **Biomarker** | **All patients AUC (95% CI)** | **ICU patients AUC (95% CI)**  **n=200** | **Normal ward patients AUC (95% CI)**  **n=240** |
| --- | --- | --- | --- |
| **penKid** | 0.645 (0.582-0.703) | 0.628 (0.531-0.716) | 0.655 (0.576-0.726) |
| **MR-proADM** | 0.627 (0.549-0.699) | 0.603 (0.499-0.698) | 0.618 (0.530-0.700) |
| **Bio-ADM** | 0.647 (0.583-0.707) | 0.620 (0.523-0.708) | 0.630 (0.545-0.708) |
| **PCT** | 0.573 (0.501-0.642) | 0.587 (0.490-0.678) | 0.548 (0.465-0.629) |
| **DPP-3** | 0.535 (0.473-0.597) | 0.602 (0.495-0.701) | 0.506 (0.447-0.566) |
| **Scr** | 0.592 (0.512-0.667) | 0.575 (0.469-0.674) | 0.595 (0.520-0.665) |

**Abbreviations:** AUC: area under the curve; AKI: acute kidney injury; ICU: intensive care unit; penKid: Proenkephalin A 119-159; MR-proADM: midregional proadrenomedullin; Bio-ADM: bioactive adrenomedullin; DPP-3: dipeptidylpeptidase-3; Scr: serum creatinine

**Table S7:** AUC of biomarkers to predict AKI within 48 h stratified by Scr values at admission (n=406)

| **Biomarker** | **AUC (95% CI)-**  **normal Scr patients (n=196)** | **AUC (95% CI)-increased Scr without CKD (n=171)** | **AUC (95% CI)- increased Scr with CKD (n=39)** |
| --- | --- | --- | --- |
| **penKid** | 0.634 (0.522-0.733) | 0.558 (0.459-0.653) | 0.682 (0.491-0.826) |
| **MR-proADM** | 0.565 (0.447-0.676) | 0.535 (0.429-0.639) | 0.663 (0.474-0.812) |
| **Bio-ADM** | 0.593 (0.463-0.710) | 0.558 (0.453-0.659) | 0.613 (0.426-0.772) |
| **PCT** | 0.452 (0.343-0.566) | 0.535 (0.430-0.636) | 0.676 (0.481-0.825) |
| **DPP-3** | 0.502 (0.385-0.619) | 0.516 (0.400-0.631) | 0.595 (0.406-0.759) |
| **Scr** | 0.515 (0.364-0.663) | 0.409 (0.313-0.512) | 0.411 (0.244-0.600) |

**Abbreviations:** AUC: area under the curve; Scr: serum creatinine; CKD: chronic kidney disease; penKid: Proenkephalin A 119-159; MR-proADM: midregional proadrenomedullin; Bio-ADM: bioactive adrenomedullin; DPP-3: dipeptidylpeptidase-3

**
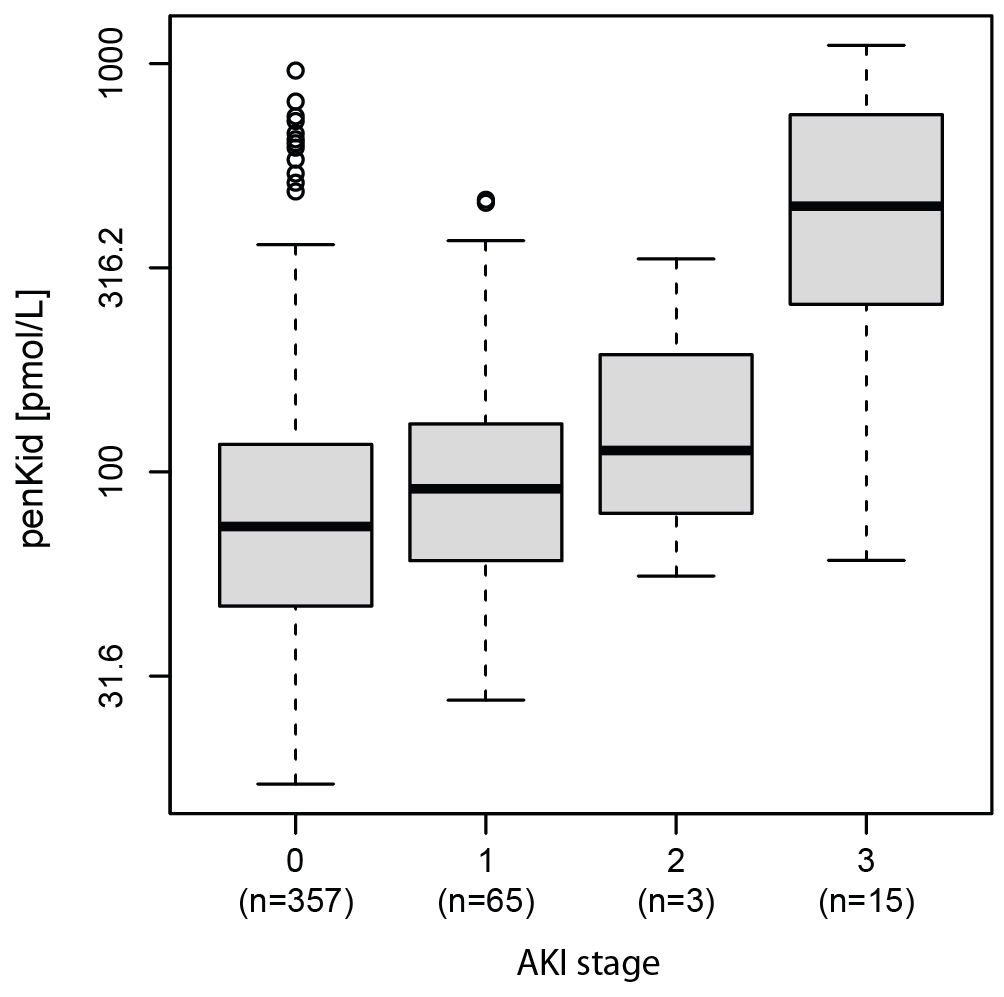
Figure S1:** penKid values depending on AKI stage. Comparison of groups using Kruskal-Wallis test with Dunn’s post-hoc test and Bonferroni adjustment indicated significant differences between stage 0 and 3 (p < 0.0001) and stage 1 and 3 (p = 0.0002).


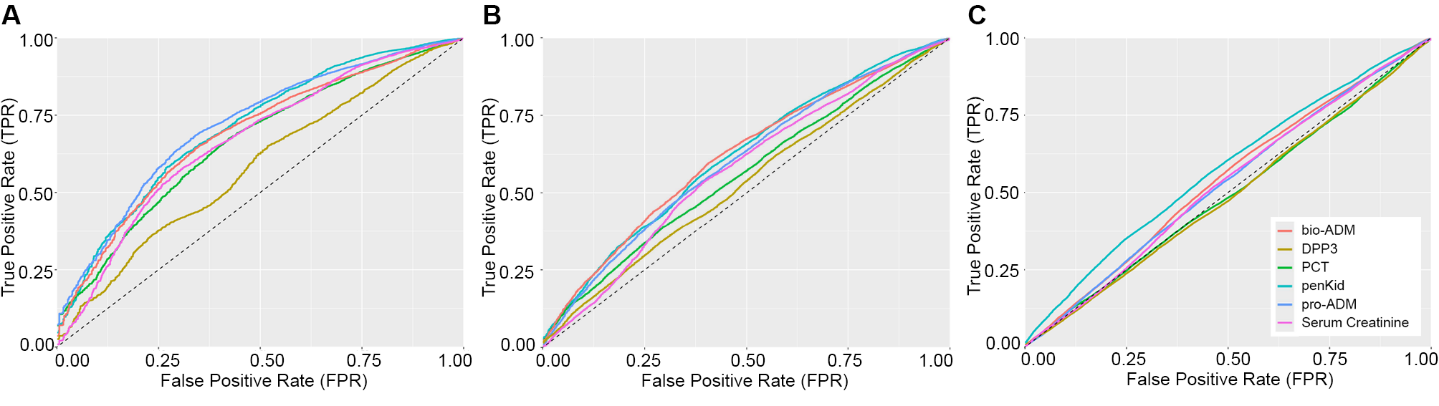


**Figure S2:** AUROC of biomarkers for prediction of AKI in the entire cohort within (**A**) 24 h (n=755), (**B**) 48 h (n=440), and (**C**) 72 h (n=328).


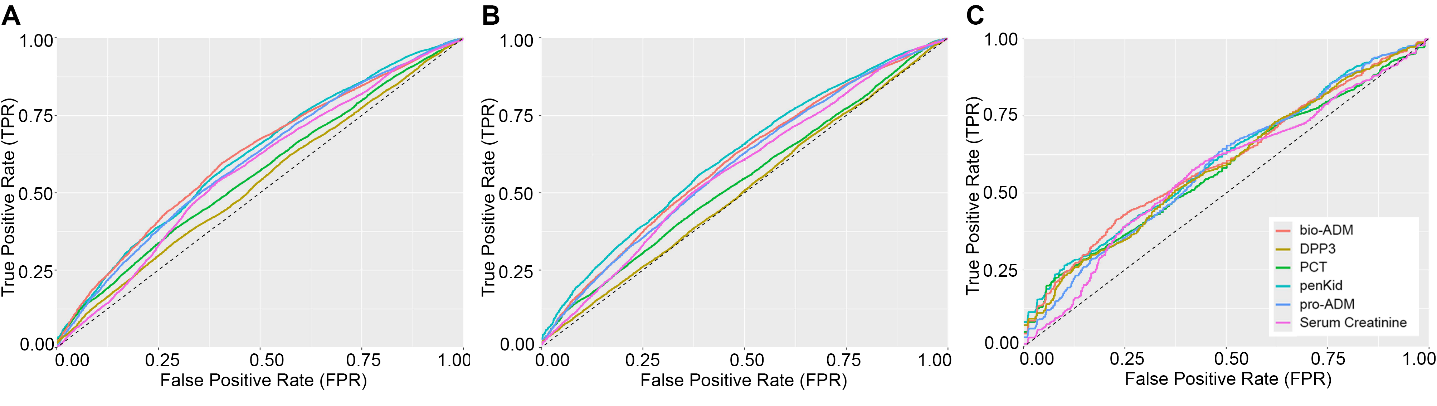


**Figure S3** AUROC of biomarkers for prediction of AKI within 48 h in (**A**) all patients (n=440), (**B**) patients transferred to normal ward (n=240), and (**C**) patients transferred to ICU within 48 h (n=200).

**
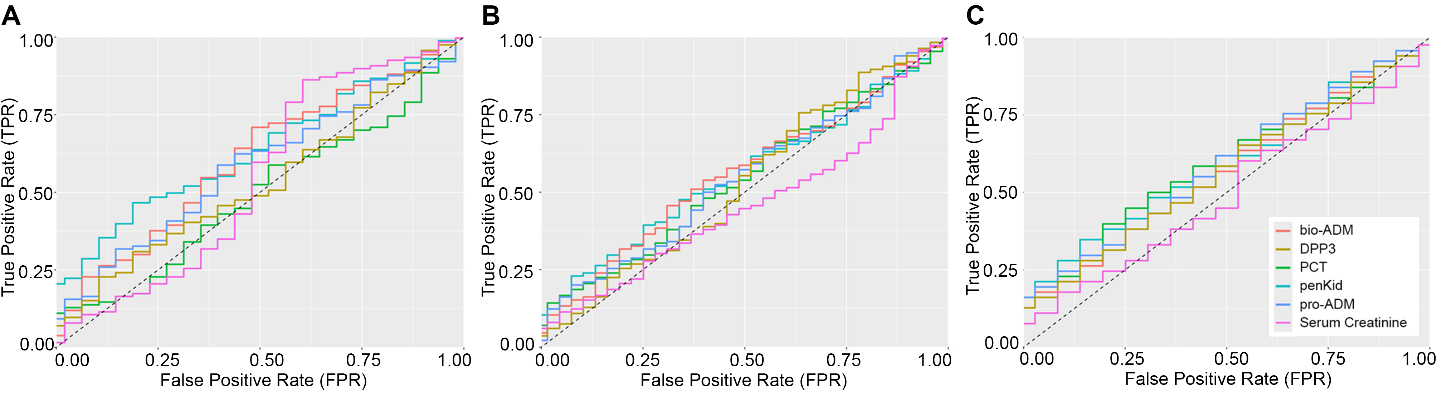
**

**Figure S4** AUROC of biomarkers for prediction of AKI within 48 h in patients with increased Scr levels with CKD (n=39).

# References

1. Bolanaki M, Winning J, Slagman A, Lehmann T, Kiehntopf M, Stacke A, Neumann C, Reinhart K, Möckel M, Bauer M. Biomarkers Improve Diagnostics of Sepsis in Adult Patients With Suspected Organ Dysfunction Based on the Quick Sepsis-Related Organ Failure Assessment (qSOFA) Score in the Emergency Department. Crit Care Med. 2024;52(6):887-99. DOI: 10.1097/CCM.0000000000006216

2. Donato LJ, Meeusen JW, Lieske JC, Bergmann D, Sparwasser A, Jaffe AS. Analytical performance of an immunoassay to measure proenkephalin. Clinical biochemistry. 2018;58:72-7. DOI: 10.1016/j.clinbiochem.2018.05.010

3. Weber J, Sachse J, Bergmann S, Sparwaßer A, Struck J, Bergmann A. Sandwich Immunoassay for Bioactive Plasma Adrenomedullin. J Appl Lab Med. 2017;2(2):222-33. DOI: 10.1373/jalm.2017.023655

4. Rehfeld L, Funk E, Jha S, Macheroux P, Melander O, Bergmann A. Novel Methods for the Quantification of Dipeptidyl Peptidase 3 (DPP3) Concentration and Activity in Human Blood Samples. J Appl Lab Med. 2019;3(6):943-53. DOI: 10.1373/jalm.2018.027995

5. Blet A, Deniau B, Santos K, van Lier DPT, Azibani F, Wittebole X, Chousterman BG, Gayat E, Hartmann O, Struck J, Bergmann A, Antonelli M, Beishuizen A, Constantin JM, Damoisel C, Deye N, Di Somma S, Dugernier T, Francois B, Gaudry S, Huberlant V, Lascarrou JB, Marx G, Mercier E, Oueslati H, Pickkers P, Sonneville R, Legrand M, Laterre PF, Mebazaa A, Adren OSSSI. Monitoring circulating dipeptidyl peptidase 3 (DPP3) predicts improvement of organ failure and survival in sepsis: a prospective observational multinational study. Crit Care. 2021;25(1):61. DOI: 10.1186/s13054-021-03471-2

6. Deniau B, Picod A, Van Lier D, Vaittinada Ayar P, Santos K, Hartmann O, Gayat E, Mebazaa A, Blet A, Azibani F. High plasma dipeptidyl peptidase 3 levels are associated with mortality and organ failure in shock: results from the international, prospective and observational FROG-ICU cohort. Br J Anaesth. 2022;128(2):e54-e7. DOI: 10.1016/j.bja.2021.11.021

7. Beunders R, Donato LJ, van Groenendael R, Arlt B, Carvalho-Wodarz C, Schulte J, Coolen AC, Lieske JC, Meeusen JW, Jaffe AS, Pickkers P. Assessing GFR With Proenkephalin. Kidney Int Rep. 2023;8(11):2345-55. DOI: 10.1016/j.ekir.2023.08.006
